# Short-term effects of low-concentration atropine eye drops on pupil size and accommodation in young adult subjects

**DOI:** 10.1007/s00417-018-4112-8

**Published:** 2018-08-25

**Authors:** Hakan Kaymak, Andreas Fricke, Yvonne Mauritz, Anne Löwinger, Karsten Klabe, Detlev Breyer, Achim Lagenbucher, Berthold Seitz, Frank Schaeffel

**Affiliations:** 1 Internationale Innovative Ophthalmochirurgie GbR, c/o Breyer Kaymak and Klabe Augenchirurgie, Martin-Luther-Platz 22, 40212 Düsseldorf, Germany; 2 Department of Ophthalmology, Saarland University Medical Center, Kirrbergerstr. 100, Gebäude 22, 66424 Homburg, Germany; 3 Institute of Experimental Ophthalmology, Saarland University Medical Center, Kirrbergerstr. 100, Gebäude 22, 66424 Homburg, Germany; 4 Eberhard Karls Universität Tübingen, Section of Neurobiology of the Eye, Institute for Ophthalmic Research, Centre for Ophthalmology, University of Tübingen, Elfriede-Aulhorn-Strasse, 772076 Tübingen, Germany

**Keywords:** Myopia, Atropine, Eye drops, Accommodation, Pupil size

## Abstract

**Purpose:**

A single eye drop containing 0.01% atropine every evening has previously been found to inhibit myopia progression in young adults. We have tested the short-term effects of very low-dose atropine eye drops on pupil sizes and accommodation in young adult subjects.

**Methods:**

Fourteen eyes of young adult subjects participated in the clinical observation. A single eye drop was applied with concentrations of either 0.01%, 0.005%, or 0.001% in the evening. Baseline parameters were measured before atropine application. Changes of pupil sizes, under photopic and mesopic conditions, as well as accommodation amplitudes were observed over the next day and analyzed by paired the Wilcoxon signed-rank test.

**Results:**

The pupil was significantly dilated 12 h after instillation of 0.01% atropine eye drops, both under photopic (3.3 ± 0.5 mm vs. 4.9 ± 0.9 mm) and mesopic (4.8 ± 0.7 mm vs. 6.1 ± 0.7 mm) conditions. Pupil sizes recovered over the day but were still significantly larger in the evening, compared to the baseline parameters measured on the day before (3.9 ± 0.5 mm vs. 5.3 ± 0.6 mm). The subjective near point of accommodation was reduced from 8.0 ± 2.4 to 6.6 ± 2.8 dpt in the morning and to 7.0 ± 2.9 dpt in the evening. At 0.005%, the pattern of results remained still similar, although the magnitude of the effects was generally smaller. At 0.001%, pupil sizes were still weakly significantly larger in the morning.

**Conclusions:**

At a dose of 0.01%, clinically significant short-term effects were detected on pupil size and accommodation for at least 24 h. At the lowest dose of 0.001%, only tiny effects on pupil size were detectable.

## Introduction

Myopia is on the rise in many industrialized countries [1–3], and it has been predicted that almost half of the world population may be myopic in the middle of twenty-first century [4, 5]. For low degrees of myopia, vision remains normal when optical corrections are applied but higher degrees of myopia carry the risk of chorioretinal complications such as macular degeneration and retinal detachment. Furthermore, the risk of cataracts and glaucoma is elevated. Based on these complications, myopia is the second most reason for blindness in mid-aged people. These complications emerge at a lifetime of the top of professional careers, about 20 years before age-related macular degenerations come into play. Based on predictions by Holden et al. [5], up to 20% of young Asian people may have high myopic progression until 2050. This would be at least 200 million people just only in the People’s Republic of China. Therefore, intervention strategies against myopia progression in children and young people must be developed. If myopia progression can be reduced, less myopia will emerge in adulthood and the risk of complication will accordingly be lower.

Several strategies are currently used to inhibit myopia progression: first, for more than 10 years, it has been known that more extensive exposure to outdoor lighting delays the onset of myopia in children [6, 7]. Later onset converts into lower end-point myopia and will therefore reduce the frequency of long-term complications. So, in Taiwan (Republic of China), children are officially encouraged by the Ministry of Education to spent 2 h per day outdoors and epidemiological data from the island actually suggest that the increase of myopia rates at first grade has been reversed [8, 9]. Second, a large body of experiments in animal models has demonstrated that emmetropization is controlled by defocus imposed on the retina in the periphery of the visual field. In rhesus monkeys, it was found that myopia can be experimentally induced with diffusers in front of the eye with a hole in the center which permitted normal vision in the fovea but degraded the image just only in the periphery [10]. Based on these results, companies (Zeiss Vision: “MyoVision”; Essilor: “Myopilux”; Cooper Vision: “MySight”) developed spectacle or contact lenses that provide full optical correction in the fovea but left the peripheral refraction more myopic. Myopic defocus typically reduces eye growth in animal models, and it is expected that myopia progression in children could be reduced. While the effects on myopia progression were generally not as powerful as hoped, however, it is proven the concept principally works. Nevertheless, there is need for more research with customized glasses. Another approach is the application of multifocal contact lenses that appeared most promising recently [11–14].

Third, atropine regained interest [15]. Atropine had been recognized as a drug against myopia already in the mid of the nineteenth century [16]. However, side effects of atropine were too severe to achieve general acceptance as a drug against myopia. Children developed photophobia, and even worse, they needed reading glasses at school. Furthermore, it was particularly discouraging that there was a strong rebound effect after termination of atropine treatment with 1% solution after 2 years [17, 18]. Myopia progression accelerated severely and reached baseline myopia of the vehicle-treated children between 3 and 4 years after beginning of atropine treatment. However, new hope was raised when it was found that atropine also reduced myopia progression at much lower dosages. At a concentration of 0.01% atropine, myopia was not inhibited in the first 3 months but then atropine started to work. After 2 years, myopia progression declined by half. Even more promising was the observation that no rebound effect occurred after termination of the treatment. Even after 5 years, the previously atropine-treated children were only about half as myopic as the controls treated with vehicle [19]. Furthermore, the side effects of 0.01% atropine were low. Pupil size was about 1 mm larger, and accommodation was only slightly reduced so that reading and near work were still easily possible without reading aids. Atropine is generally assumed to inhibit myopia through its action in the retina and choroid and, perhaps, in the sclera [20, 21].

Based on these findings, atropine underwent a revival also in Europe, including Germany [22–24]. It is used off-label. This means that the diluted atropine solutions are not normalized and vary from place to place. It has also not been evaluated whether even lower concentrations may still be effective. As a first step, we have therefore studied how atropine eye drops at a dose of 0.01%, 0.005%, and 0.001% affect pupil sizes and accommodation amplitudes in young subjects. Furthermore, it was studied how long these effects persist after single binocular application.

## Methods

### Subjects

Twenty eyes of young adult subjects (28.4 ± 5.8 years; range 21–40 years) with no ocular pathologies other than moderate refractive errors (− 0.7 ± 0.9 dpt; − 3.1–0.6 dpt) were recruited by the physicians of the clinics. In the context of our quality management, all probands were informed about side effects of atropine eye drops and signed an informed consent.

### Procedures

Baseline parameters of pupil size, pupil dynamics, and accommodation amplitudes were recorded in each subject by a kerato-refractometer (Aladdin Biometer; Topcon Europe Medical BV, Capelle aan den IJssel, Netherlands). The subjective near point of accommodation was determined by orthoptists.

A single atropine eye drop was applied in the evening of the day before of the observation, either at a concentration of 0.01% or 0.005% or 0.001%. Pupil sizes and pupil dynamics, as well as accommodation amplitudes, were compared in the morning (8:00–11:00 a.m.), at around noon (11:40 a.m.–3:00 p.m.), and in the late afternoon (3:00 p.m.–6:30 p.m.).

### Atropine solutions

Atropine eye drop solution in one-dose ophtioles was aseptically manufactured by an external apothecary (Berg-Apotheke, Tecklenburg, Germany). The solution was stable for 1 year, if stored refrigerated. Five milliliters of atropine solution was prepared from 0.0005 g atropine sulfate, 0.5 g of 0.02% thimerosal stock solution, and 0.0925 g boric acid and filled up with 4.407 g aqua. The eye drop size was about 60 μl, as calculated from the number of drops that could be delivered by a 0.5-ml vial at room temperature. Accordingly, one eye drop of the 0.01% solution contained 0.0005 × 600 / 5000 = 60 μg atropine.

### Statistics

To evaluate potential differences in short-term effects of a low concentration of atropine eye drops on pupil size and accommodation in young adult subject, statistical comparison was performed by the two-sided Wilcoxon signed-rank test (OriginPro 2017 SR1, OriginLab Corporation, Northampton, MA, USA). The criterion for statistical significance was *p* ≤ 0.5.

## Results

### Effects of atropine on pupil sizes

**Fig. 1:**
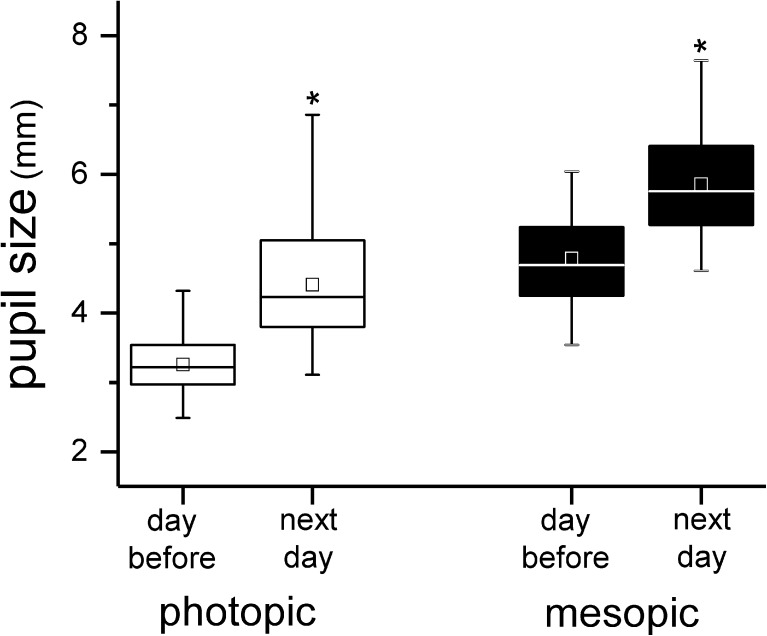
Pupil sizes on the day before and the day after instillation of 0.01% atropine eye drops under photopic and mesopic conditions. The edges of boxes indicate 25th percentile, median, and 75th percentile, and whisker displays the minimal and maximal values. *p ≤ 0.001, statistically significant differences to pupil size without atropine eye drops

Even 0.01% atropine solution had a conspicuous effect on pupil sizes, no matter whether the comparison was done under photopic or mesopic conditions for 20 eyes (Fig. 1). The comparison refers to pupil sizes on the day before and the day after atropine application. These first measurements after atropine application were done over the whole day (8.00 a.m.–3:00 p.m.).

**Fig. 2:**
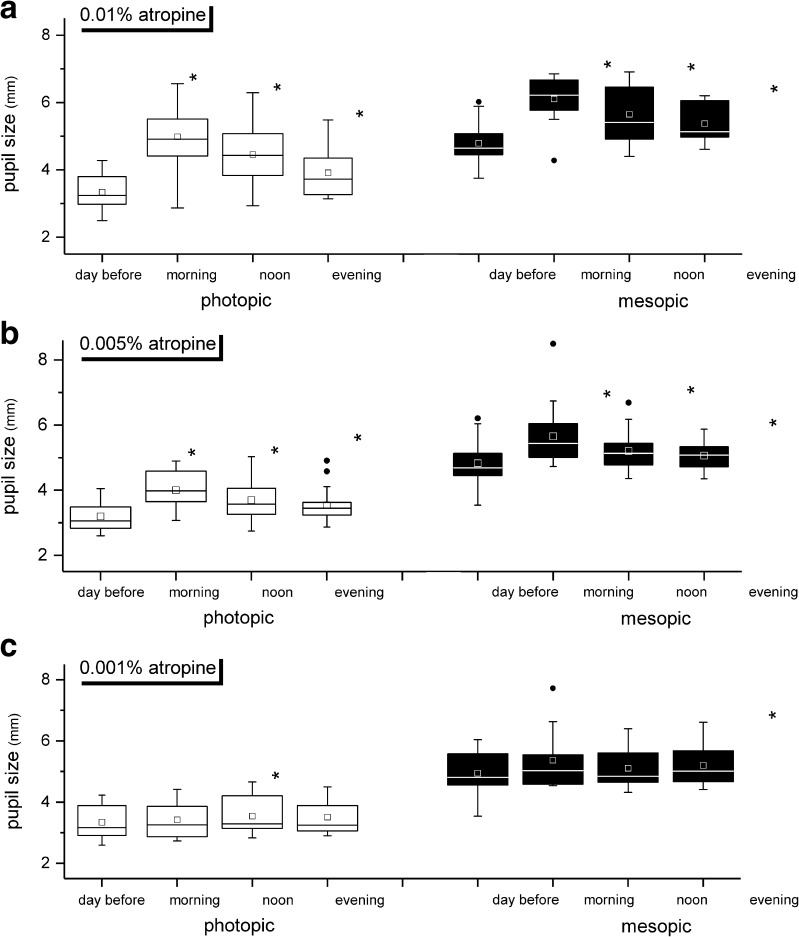
Baseline pupil sizes on the day before atropine application, on the day after application in the morning, at noon, and in the evening under photopic (left) and mesopic (right) conditions. **a** 0.01% atropine. **b** 0.005% atropine. **c** 0.001% atropine. Note that pupils were clearly dilated after atropine application in the morning of the observation day both at 0.01% and, less so, at 0.005% atropine, but not with 0.001%. Note also that the effect declined about over the day (most clearly seen in **a**, photopic conditions). The edges of boxes indicate 25th percentile, median, and 75th percentile, and whisker displays the minimal and maximal values. Dots represent statistical outlier. *p ≤ 0.05, statistically significant differences to pupil size without atropine eye drops

Time-dependent short-term effects of atropine onto pupil size on the day after evening application are shown in Fig. 2. Furthermore, the mydriatic effect of atropine was also detectable at lower doses. So, the action profiles of atropine on pupil sizes over the day under photopic (left) and mesopic (right) conditions are shown in Fig. 2 for doses of 0.01% (A), 0.005% (B), and 0.001% (C) atropine.

At the highest dose (0.01%), the effects on pupil size were still significant in the evening for photopic (day before application 3.3 mm ± 0.5 mm; evening after application 3.9 mm ± 0.8 mm; *p* ≤ 0.02) and mesopic (4.8 ± 0.7; 5.4 ± 0.5; *p* ≤ 0.02) conditions. The pupil size after application of 0.005% atropine eye drops was significantly expanded at least 24 h (photopic condition 3.2 ± 0.5; 3.5 ± 0.5; *p* ≤ 0.001; mesopic condition 4.8 ± 0.7; 5.1 ± 0.4; *p* ≤ 0.001). In contrast, at the lowest used concentration of atropine (0.001%), no clinical significant dilatation was detected to any time point analyzed.

It is striking that the variability in pupil sizes clearly increased after atropine application, suggesting that atropine was differently effective in different subjects, either because drop sizes varied or because different amounts reached the targets or because subjects were differently sensitive to the effect of the anti-muscarinic agent. Also, iris pigmentation could play a role [25]. By comparing the dilatation in dependency of eye color, we found no clinically significant differences between blue and not blue eyes. For instance, at the highest dose (0.01%), the pupil size is 4.0 mm ± 0.9 mm and 3.7 mm ± 0.7 mm for blue and not blue eyes at the evening after application (each 10 eyes), respectively.

### Effects of atropine on accommodation amplitude

**Fig. 3:**
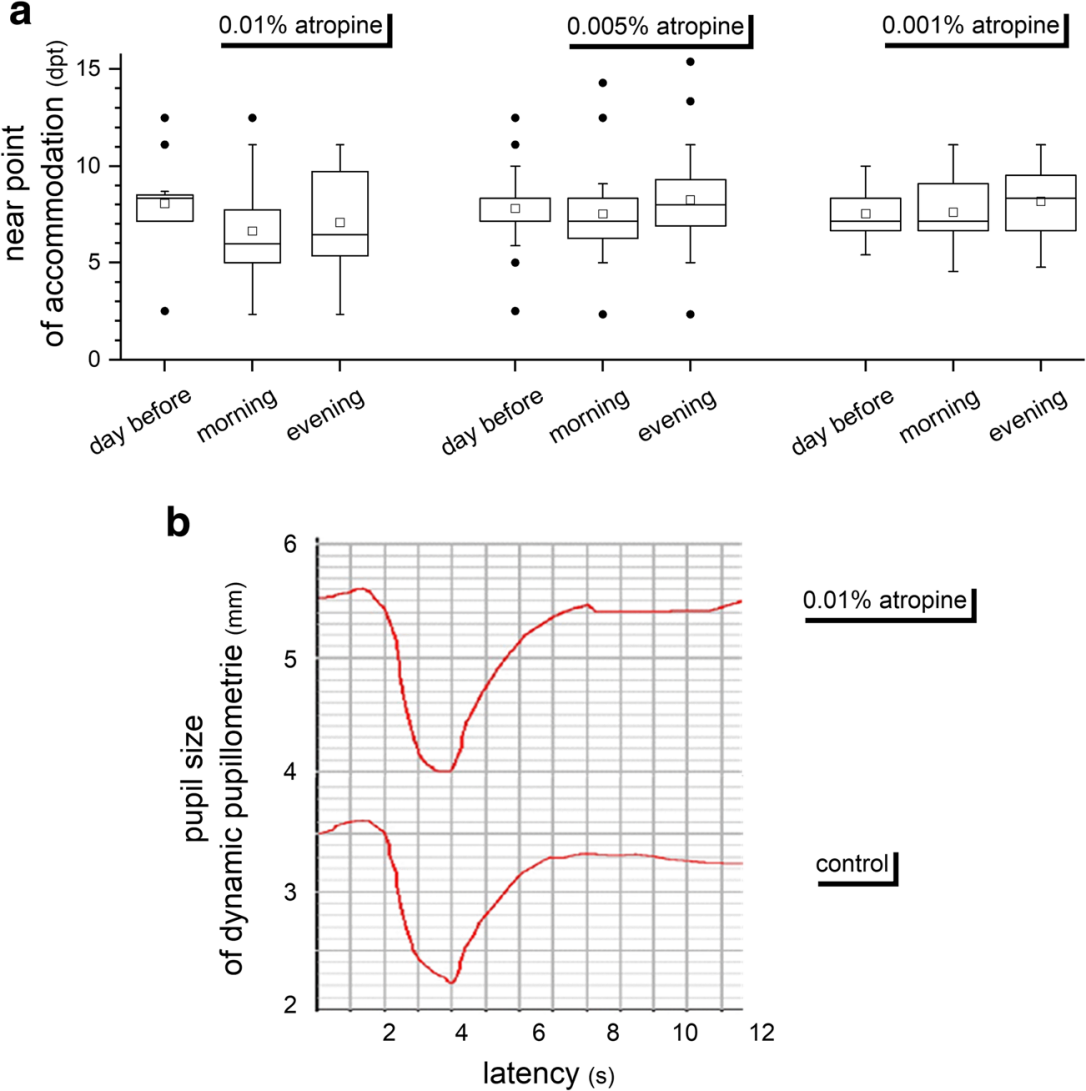
**a** Effects of low-dose atropine on near-point accommodation at doses of 0.01%, 0.005%, and 0.001% atropine. The edges of boxes indicate 25th percentile, median, and 75th percentile, and whisker displays the minimal and maximal values. Dots represent statistical outlier. *p ≤ 0.05, statistically significant differences to pupil size without atropine eye drops. **b** Exemplified dynamic pupillometry without and after application of 0.01% atropine eye drops. Procedure: 2 s mesopic, followed by 2 s photopic, followed by relaxation under mesopic conditions

Subjects experienced a minor decline of their subjective accommodation amplitude at a dose of 0.01% which achieved significance (*p* < 0.002) only in the morning. At all other doses and time points, no significant effects were found. So, at a dose of 0.01% atropine, the near point of accommodation was determined at 8.1 dpt ± 2.4 dpt on the day before and 7.1 ± 2.8 on the evening after application (*p* = 0.06). Respectively, 7.8 ± 2.0 and 8.3 ± 2.7 (*p* = 0.18) for 0.005% and 7.5 ± 1.2 and 8.1 ± 1.8 (*p* = 0.10) for 0.001% atropine were measured (Fig. 3a).

Pupil dynamics was also studied before and after atropine application. However, no significant differences in pupil amplitude and constriction or dilatation speed were observed, as exemplified in Fig. 3b.

### Diurnal variations in pupil size

**Fig. 4:**
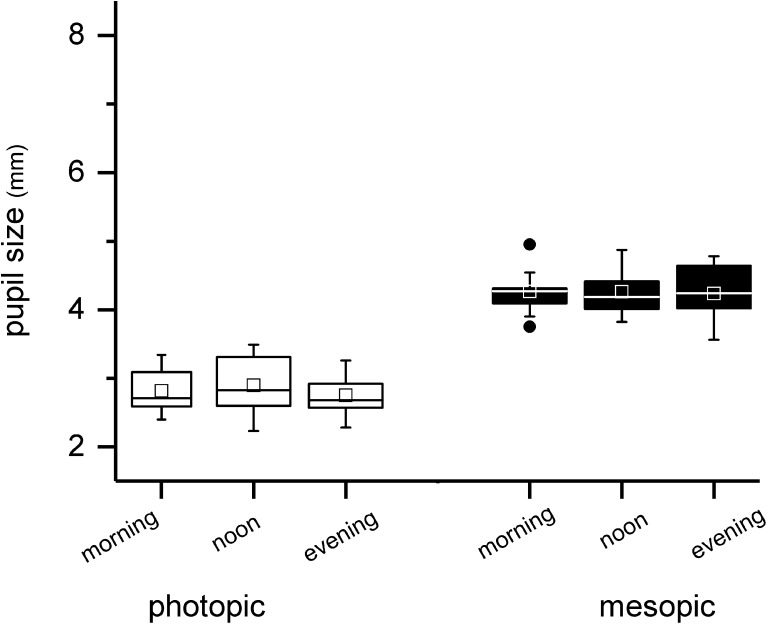
Diurnal variations in pupil size under photopic and mesopic conditions. No changes were observed over the day. The edges of boxes indicate 25th percentile, median, and 75th percentile, and whisker displays the minimal and maximal values. Dots represent statistical outliers

To exclude that the observed effects were not due to atropine but rather due to diurnal variations, pupillometric analysis was done in the morning, at noon, and in the evening, too. However, for all analyzed parameters, no significant changes were detectable. Therefore, no shift is seen in pupil size neither under photopic (morning 2.8 mm ± 0.3 mm; evening 2.7 mm ± 0.3 mm; *p* = 0.39) nor under mesopic (4.1 ± 0.7; 4.2 ± 0.4; *p* = 0.36) conditions (Fig. 4).

## Discussion

Atropine eye drops at doses of 0.01 and 0.005% had minor effects on pupil sizes. In the case of the 0.005% solution, a nearly full recovery has been observed after 24 h. With 0.01%, a minor effect on pupil size was still detectable in the evening after application (*p* < 0.03). No significant effects were detected after application of 0.001% atropine eye drops. Accommodation amplitude was slightly reduced only with 0.01% atropine and only at the first point of measurement in the morning of the same day. In summary, the effects of low-dose atropine were small in the young adult subjects and represented no drawback for a potential long-term application of atropine as an intervention against myopia progression.

Certainly, children eyes are smaller (21–22 mm at the age of 10 years, compared to 24 mm in adults).

The difference in volume is about 30% which would raise the intraocular concentration by 30% (factor 1.3). Eyes of myopic children have an axial length of 23–25 mm and, therefore, a similar volume to non-myopic adult eyes. So, the difference to young adults is still minor compared to the differences in tested doses (factor 10). It could also be the children’s corneal permeability for atropine is higher which would increase the intraocular concentrations. However, there are no data showing that this is the case. Therefore, the current data of young adult eyes should also be applicable to children’s eyes. Nevertheless, it should be kept in mind that the results presented here are based on data from 21–40-year-old subjects. Younger probands normally have a larger range of accommodation and a larger pupil size, so the results found cannot be transferred directly one-to-one to children’s eyes.

Furthermore, it should be noted that the results described here were carried out with atropine eye drops with the preservative thimerosal [26]. The preservative should cause an increase in permeability of the cell membranes. Therefore, it must be assumed that the short-term effect of atropine eye drops used is greater than eye drops without thimerosal.

### Increased variability of pupil sizes after atropine application

A striking observation was that the effects of atropine on pupil sizes were variable among subjects. Standard deviations of pupil sizes clearly increased. For instance, under photopic condition and application of 0.01% atropine eye drops, the pupil size was dilated from a mean of 3.3 mm ± 0.5 mm (range 2.5 mm–4.3 mm) to a mean of 5.0 mm ± 0.8 mm (range 2.9 mm–6.3 mm) in the morning (Fig. 1). This could indicate that eye drop volumes varied, that penetration of atropine through the cornea and diffusion through the tissues varied, or that subjects were differently sensitive to muscarinic antagonists. Furthermore, it could be that iris pigmentation affects atropine diffusion. While the first and the last possibility can be easily tested, the other two are hard to prove without using radio-labeled atropine molecules and measure the binding curves, as has been done in tree shrews by Vessey et al. [27]. The large variability of the atropine effects should be kept in mind when individually different potency of atropine is observed during myopia inhibition in children.

### Time constants of atropine action and potential accumulation during repeated application

It is remarkable that the mechanisms of myopia inhibition by atropine are still not clear. An accommodation-related mechanism has been excluded [21]. The effects of atropine in the retina share features of light stimulation since both nitric oxide and dopamine production and release are stimulated [28]. Atropine also increases contrast sensitivity at intermediate spatial frequencies in both chicks and mice [29–31]. It is unlikely, however, that atropine acts on myopia through muscarinic receptors [20, 28]. A major argument in support of this view is that the tissue concentrations necessary to achieve myopia suppression are much higher than what would be needed to half-saturate muscarinic receptors. Furthermore, it binds also to alpha-adrenergic receptors with similar binding constants, and some adrenergic antagonists also inhibit myopia in chicks. It is therefore of interest to estimate the fundal concentrations of atropine when a single eye drop is given that contains 60 μg atropine. We could not find any publications in which the transcorneal penetration of atropine sulfate has been measured. Assumed that 10% of the atropine supplied by the eye drops penetrates through the cornea and the intraocular volume is 7.2 cm^3^ (7.2 ml), the vitreal concentration would be about 0.83 ng/μl. With a molecular weight of atropine sulfate of 694.8 g, this converts into a solution of 11.9 nM. The M4 receptor half-saturation concentration for atropine is 823 pM or 0.823 nM. This suggests that about 15 times more atropine sulfate molecules are available when eye drops of 0.01% concentration are used. If the dose is further reduced to 0.005% or 0.001%, one approaches the receptor half-saturation constant, always assuming that, in fact, one tenth of the topically applied atropine really reaches its fundal targets. Another issue is whether the atropine concentrations can accumulate with daily applications. Since the effects on pupil size of 0.01% atropine solution were still detectable in the evening of the day in the present study, some atropine must still have been present. The remaining amount could add to the atropine applied during repeated instillation, and it would be of interest to find out whether pupil sizes further increase during prolonged application. In the case of even lower doses (0.005 and 0.001%), accumulation appears unlikely, but this could also be tested by studying pupil behavior during a sequence of repeated daily applications.
